# Intermittent Preventive Treatment with Sulfadoxine–Pyrimethamine: More than Just an Antimalarial?

**DOI:** 10.4269/ajtmh.16-0888

**Published:** 2017-01-11

**Authors:** Julie Gutman, Laurence Slutsker

**Affiliations:** 1 Malaria Branch, Division of Parasitic Diseases and Malaria, Centers for Disease Control and Prevention, Atlanta, Georgia.; 2 Malaria and Neglected Tropical Diseases, Center for Malaria Control and Elimination, PATH, Seattle, Washington.

Malaria in pregnancy is associated with increased risk for both maternal and neonatal adverse outcomes, notably low birthweight and neonatal mortality.^1^ Since 2004, following studies that showed that intermittent preventive treatment with sulfadoxine–pyrimethamine (IPTp-SP) reduced the risk of these adverse events,^2^ the World Health Organization (WHO) recommended IPTp-SP for all areas in Africa with moderate-to-high malaria transmission.^3^ IPTp-SP is associated with significant reductions in low birthweight, with a protective efficacy of approximately 26% in an analysis of national survey data from 32 countries.^4^ This impact has been presumed to be a result of the antimalarial effects of SP. Because resistance to SP has increased, particularly in eastern and southern Africa, SP is no longer recommended for treatment of acute malaria illness, even in combination with artemisinins. Despite this, even in areas where the efficacy of SP to clear parasitemia has clearly decreased, IPTp-SP has continued to show benefit for preventing low birthweight.^5^ Moreover, no other antimalarials have yet been shown to be an ideal replacement for SP for IPTp. Studies evaluating potential alternative IPTp regimens have had mixed outcomes on birthweight,^6^,^7^ leading to the hypothesis that SP may exert some of its effect through antibacterial or anti-inflammatory actions.^6^ In Lusaka, Zambia, where malaria parasite prevalence is < 1%, Stoner and others show that among human immunodeficiency virus (HIV)–positive women, receipt of IPTp-SP was associated with a dose-dependent reduction in the risk of low birthweight, as well as an increase in gestational age, further suggesting a mechanism other than antimalarial activity as an explanation for a reduced risk of low birthweight among women receiving IPTp-SP during pregnancy.^8^

The current WHO recommendation is for all HIV-positive women to receive daily co-trimoxazole (an antifolate antibiotic that is similar to, but shorter acting than SP) for prevention of opportunistic infections. The co-administration of co-trimoxazole and SP is contraindicated because of an increased risk of adverse effects. Co-trimoxazole has antimalarial activity; in one study, infant birthweight was similar among HIV-positive women taking daily co-trimoxazole and those taking IPTp-SP (3-dose goal).^9^ In a multicenter trial assessing the benefit of adding IPTp with mefloquine to daily co-trimoxazole in HIV-infected women, mefloquine significantly reduced maternal peripheral and placental malaria infection at delivery, but did not have a differential impact on birthweight compared with women taking daily co-trimoxazole alone, again suggesting an effect on birthweight independent of antimalarial activity.^10^

That SP, which has antibacterial activity, could improve birthweight independent of its antimalarial effect is perhaps unsurprising. A non-malaria-related beneficial effect of SP on birthweight could be due to several mechanisms, including anti-inflammatory effects or alterations in the bacterial flora of the gut or vagina leading to effects on maternal or infant weight gain, indirect metabolic effects, or a reduction in the impact of genitourinary tract organisms associated with adverse pregnancy outcomes.^11^ Though the mechanisms behind these benefits remain unclear, farmers have long used low doses of antibiotics to fatten farm animals, and a recent study in humans found an association between early infant antibiotic use and increased body mass index.^12^ A study in mice found that antibiotic-induced changes in the intestinal microbiome led to alterations in lipid and cholesterol metabolism, with resulting increases in adiposity^13^; whether this is the case in humans remains to be seen. Alternately, as the authors hypothesize, SP could be treating or preventing other infections,^14^ thus preventing preterm delivery and associated low birthweight.

What is more surprising is that SP seemed to counteract the effects of antiretrovirals on pregnancy outcomes. Although administration of antiretroviral therapy (ART) to pregnant women, compared with not treating mothers, is associated with improved birthweight,^15^ and is clearly to the benefit of both mothers and infants, concerns remain about the safety of ART in pregnancy. As in other studies,^16^,^17^ Stoner and others found an association between ART and increased risk for low birthweight and preterm delivery.^8^ The reasons for this remain unclear, but may include modulation of the normal immune shift from Th1 to Th2 that occurs during pregnancy, other alterations in inflammatory cytokines, or possibly an increased risk of hypertension with resultant placental insufficiency.^16^ The mechanisms by which SP might counteract this effect are unclear, and more research is needed to understand the underlying mechanisms by which ART might increase risk for low birthweight, and whether this occurs with all combinations or only with specific drugs, combinations, or drug classes. In this study, despite adjusting for CD4 count in the analysis, the association between ART and low birthweight may still have been simply related to the fact that women with more advanced HIV were more likely to be on ART, as the study used data from the era before widespread implementation of the recommendation for all pregnant women with HIV, regardless of CD4 count, to be started on combination ART as soon as diagnosed, and continued for life (Option B Plus).

In light of concerns about the decreasing antimalarial efficacy of SP, recent studies have explored alternatives for IPTp, including dihydroartemisinin–piperaquine (DP), with demonstrated protection against malaria, but no clear benefit compared with SP with respect to birthweight.^6^,^7^ Given the inconsistent evidence of improved birth outcomes with the use of IPTp-DP, the WHO recommended further study into both the efficacy and feasibility of IPTp-DP to inform a future recommendation (http://www.who.int/malaria/publications/atoz/istp-and-act-in-pregnancy.pdf). The findings of Stoner and others provide evidence that in a setting where malaria was not likely an important factor affecting pregnancy outcome, IPTp-SP still appeared to confer benefit through as yet undefined pathways. Further research is needed to elucidate these other mechanisms, as well as to explore whether daily co-trimoxazole confers similar benefit.
